# Genetics redraws pelagic biogeography of *Calanus*

**DOI:** 10.1098/rsbl.2017.0588

**Published:** 2017-12-20

**Authors:** Marvin Choquet, Maja Hatlebakk, Anusha K. S. Dhanasiri, Ksenia Kosobokova, Irina Smolina, Janne E. Søreide, Camilla Svensen, Webjørn Melle, Sławomir Kwaśniewski, Ketil Eiane, Malin Daase, Vigdis Tverberg, Stig Skreslet, Ann Bucklin, Galice Hoarau

**Affiliations:** 1 Faculty of Biosciences and Aquaculture, Nord University, 8049 Bodø, Norway; 2 Department of Arctic Biology, The University Centre in Svalbard, N-9171 Longyearbyen, Norway; 3 Institute of Oceanology, Russian Academy of Sciences, 117997 Moscow, Russia; 4 Department of Arctic and Marine Biology, UiT-the Arctic University of Norway, 6050 Tromsø, Norway; 5 Institute of Marine Research, 5005 Bergen, Norway; 6 Institute of Oceanology, Polish Academy of Sciences, 81-712 Sopot, Poland; 7 Department of Marine Sciences, University of Connecticut Avery Point, Groton, CT 06340, USA

**Keywords:** zooplankton, genetics, climate change, species identification, fjord, ecosystem shift

## Abstract

Planktonic copepods of the genus *Calanus* play a central role in North Atlantic/Arctic marine food webs. Here, using molecular markers, we redrew the distributional ranges of *Calanus* species inhabiting the North Atlantic and Arctic Oceans and revealed much wider and more broadly overlapping distributions than previously described. The Arctic shelf species, *C. glacialis*, dominated the zooplankton assemblage of many Norwegian fjords, where only *C. finmarchicus* has been reported previously. In these fjords, high occurrences of the Arctic species *C. hyperboreus* were also found. Molecular markers revealed that the most common method of species identification, prosome length, cannot reliably discriminate the species in Norwegian fjords. Differences in degree of genetic differentiation among fjord populations of the two species suggested that *C. glacialis* is a more permanent resident of the fjords than *C. finmarchicus*. We found no evidence of hybridization between the species. Our results indicate a critical need for the wider use of molecular markers to reliably identify and discriminate these morphologically similar copepod species, which serve as important indicators of climate responses.

## 1. Introduction

**Figure 1.**
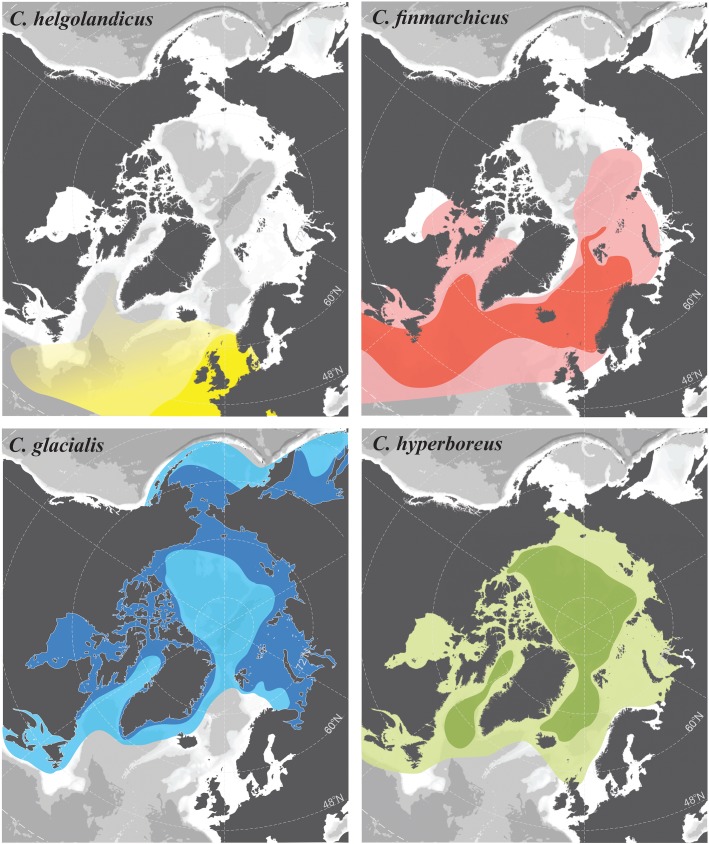
*Calanus* species distributional ranges in the North Atlantic and Arctic Oceans based on morphological identification from previous studies (sources in electronic supplementary material, S8). For each panel, dark-shaded colour represents core area for each species, where reproduction is known to occur; light-shaded colour represents the total described distributional area.

Copepods of the genus *Calanus* are central in North Atlantic and Arctic pelagic food webs. Rich in lipids, they form a key link between primary producers and secondary consumers and predators. Four species of the genus *Calanus* occur throughout the North Atlantic and Arctic Oceans (figure 1): *C. helgolandicus* (*Chel*), *C. hyperboreus* (*Chyp*), *C. finmarchicus* (*Cfin*) and *C. glacialis* (*Cgla*); and there has been considerable effort to document and model their distributional changes [1,2]. Importantly, abundances and dynamics of fish stocks are strongly associated with *Calanus* species composition and abundances [3], and climate-driven changes in their biogeographical distributions (i.e. range shifts) can lead to ecosystem regime shifts and potential collapse of fish stocks such as cod [4]. However, distinguishing *Calanus* species is challenging due to their morphological similarity and lack of diagnostic characters. The usual method of species identification is body (prosome) length, although this approach has been questioned [5,6]. Misidentification may thus occur, impacting the reliability of our current knowledge of species distributions and preventing accurate assessment of species geographical range shifts in response to climate change.

Here we re-examine the distributional ranges of four co-occurring *Calanus* species in the North Atlantic and Arctic Oceans, using six molecular markers designed to ensure reliable species identification.

## 2. Material and methods

### (a) Sample collection

Zooplankton samples were collected from 83 locations in the North Atlantic and Arctic Oceans (electronic supplementary material, S1) by vertical nets tows with 150–200 µm mesh sizes and preserved in 70–80% ethanol. A Folsom plankton splitter was used to make subsamples containing up to 150 *Calanus* individuals from developmental stage CIV–CVI (electronic supplementary material, S1). No species level morphological identification was performed for any individuals.

### (b) Molecular species identification

DNA was extracted from the excised antennae of each specimen using the HotSHOT protocol [7], and molecular species identification of 4434 individuals was achieved using six nuclear markers type InDels (Insertion or Deletion motifs) [8] scored on a 3500xL genetic analyzer (Applied Biosystems). These bi-parentally inherited markers are easy to use and can potentially detect hybridization [9]. Their reliability was confirmed by the traditional, but more cost- and labour-intensive mitochondrial 16S rDNA sequencing (mtDNA) [10,11] of 159 individuals from 53 locations (electronic supplementary material, S2 and S3), following Smolina *et al*. [8]. In addition, 129 individuals from Saltfjord/Skjerstadfjord were measured (prosome length) and sequenced for the 16S (table 1; electronic supplementary material, S4 and S5). Identification of specimens from InDels and 16S rDNA sequences was congruent for all 677 individuals investigated (288 in present study (electronic supplementary material, S2–S4) and 389 in Nielsen *et al*. [9]). InDel markers were also used to test for the presence of hybrids between *Cfin* and *Cgla* [9] (electronic supplementary material, S6).

**Table 1.** Comparison of *Calanus finmarchicus* (*Cfin*) and *C. glacialis* (*Cgla*) identification methods in Saltenfjord/Skjerstadfjord.

				prosome length range (μm)			
Saltenfjord/Skjerstadfjord	InDel species ID	16S rDNA species ID	markers' congruence	*N*	stage CV	*N*	stage CVI female
*Cfin*	89	89	100%	26	1976.64–2717.76	14	2406.89–2747.02
*Cgla*	40	40	100%	20	2119.40–2623.33	69	2150.68–3030.50

### (c) Population differentiation

Population genetic analysis was carried out to distinguish between fjord resident and drifting (seasonally transient) species [12] (electronic supplementary material, S7). Focusing on *Cfin* and *Cgla* populations, genetic differentiation was measured using the global index of population differentiation, *F*_ST_ [13], based on 10 microsatellite DNA markers [14,15] assayed for 24 individuals per species from three locations: Isfjord, Saltfjord and Lurefjord.

## 3. Results and discussion

**Figure 2.**
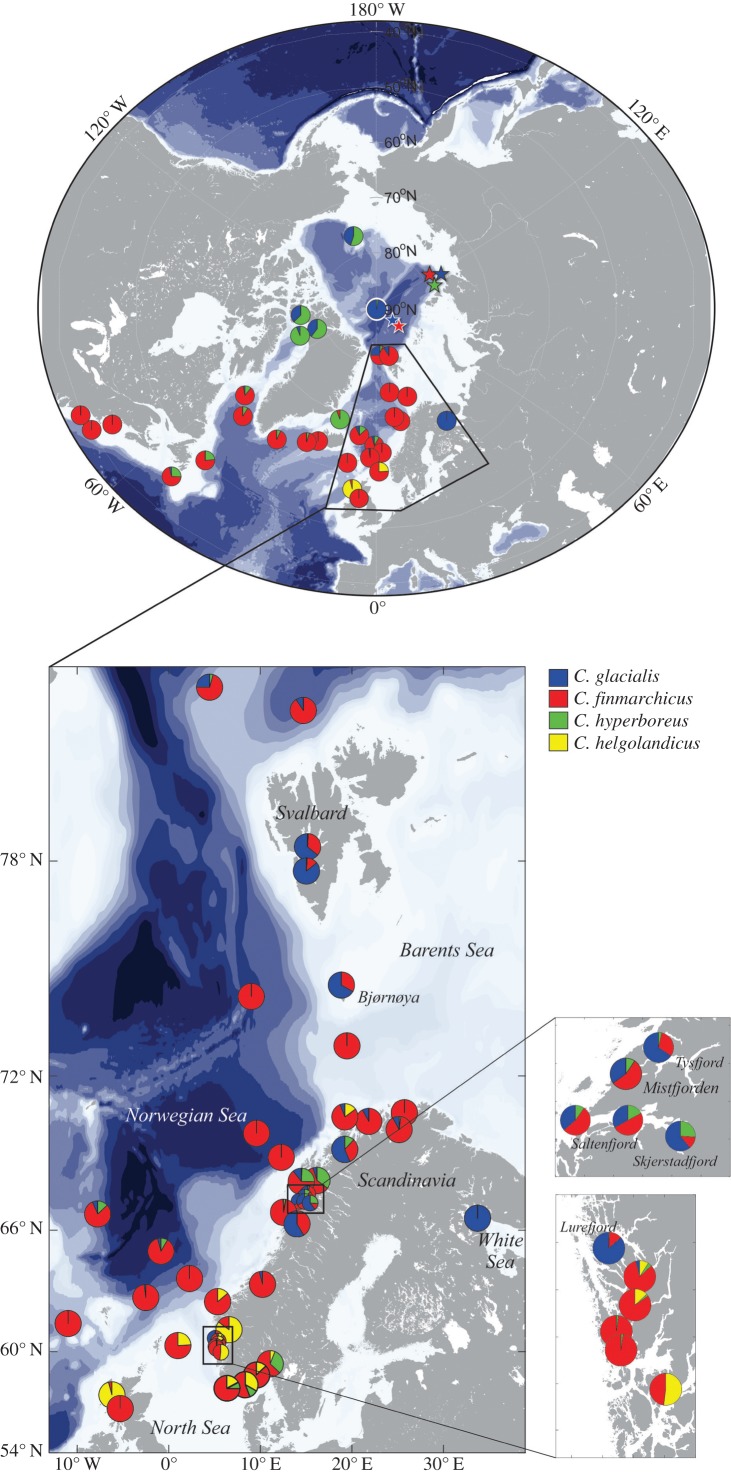
*Calanus* species distributional ranges in the North Atlantic and Arctic Oceans based on molecular species identifications. Pie charts represent relative frequencies of *C. glacialis* (blue), *C. finmarchicus* (red), *C. hyperboreus* (green) and *C. helgolandicus* (yellow) in each sample. Stars indicate non-quantitative species records.

Identification of *Calanus* species using molecular markers revealed that all four species have much wider distributional ranges than previously reported (figures 1 and 2; electronic supplementary material, S1), as suggested by an earlier molecular study [6]. The distribution of *Chel* was known to extend from the Mediterranean Sea to the North Sea (58° N, figure 1) [16]. Here, we identified *Chel* in several Norwegian fjords and in the Norwegian Sea as far north as 70**°** N (figure 2). Specimens found in Myken stations (66° N) and near Tromsø (70° N) could result from transport in ocean frontal jet currents running from the North Sea along the Norwegian coast. However, the high prevalence (85%) of the species recorded in the relatively isolated Sognefjord (61° N) may represent a locally established population. It remains to be tested whether *Chel* has always been present in these fjords but misidentified, or whether our findings represent evidence of a recent biogeographical range shift.

Previous reports of the Arctic *Chyp* [17] occurring in the northern Norwegian Sea (figure 1) have been attributed to transport of individuals by Arctic intermediate waters [18]. Here, we detected the species in large proportions along the Norwegian coast, everywhere north of 58° N (figure 2; electronic supplementary material, S1). Whether the southern presence of *Chyp* results from advection from Arctic stocks or from self-reproducing populations remains to be investigated.

*Calanus finmarchicus* is currently considered to be an indicator species of North Atlantic water masses [17], and our results largely support this view (figure 2). The genetically confirmed species range extends as far north as 87° N and as far east in the Arctic as the eastern boarder of the Laptev Sea (78° N, 113° E, figure 2), regions of the Arctic Ocean affected by Atlantic inflow. It was proposed that *Cfin* may thrive in these Northern regions and replace *Cgla* in response to Arctic warming [19], however, at present the individuals recorded at these most northerly locations were likely transported from southern populations [19].

*Calanus glacialis* is regarded as a true Arctic shelf species, which serves as a circumpolar indicator of these waters [17] (figure 1). We rarely observed it offshore in Atlantic waters, but documented the species' occurrence in many Norwegian fjords, as far south as 60° N (figure 2), where it usually co-occurred with *Cfin* in fjords with deep basins separated from shelf waters by shallower sills (electronic supplementary material, S1). In several fjords, *Cgla* dominated over other *Calanus* species; we recorded a positive gradient of relative abundance of *Cgla* from the mouth to the innermost areas of some fjords (e.g. Ranfjord, electronic supplementary material, S1).

In the fjords, prosome length of *Cgla* and *Cfin* overlapped completely (table 1; electronic supplementary material, S5), which explains why *Cgla*'s large occurrence has not been reported previously. Furthermore, recent work by our group shows that morphological characters cannot reliably distinguish between *Cfin* and *Cgla* throughout their range [20].

Some zooplankton species are long-term residents of Norwegian fjords, while others are replaced periodically with basin water exchanges [21]. Resident species are expected to show greater genetic differentiation among fjord populations than drifting species [12]. Our analysis found no significant genetic differentiation among fjord populations of *Cfin* (*F*_ST_ = 0.004^n.s.^), but *Cgla* populations did differ significantly (*F*_ST_ = 0.03*), suggesting lower rates of exchange (i.e. gene flow) for *Cgla* than for *Cfin.* These results support previous descriptions of *Cfin* as a drifting species [12] that is advected into and out of fjords seasonally [22]. Less gene flow—together with the absence of offshore populations—suggests that *Cgla* populations are resident [12]. In both the White Sea [23] and Lurefjord [24], *Cgla* is known to migrate in early summer from warm surface layers to colder deep water. This may explain the species' ability to maintain local populations and avoid transport out of fjords.

Hybridization between *Cfin* and *Cgla* has been suggested in the Northwest Atlantic [14] based on microsatellite markers developed for *C. finmarchicus*. Notably, no first-generation hybrids were found in our survey of 4434 individuals from samples collected throughout the Northeast Atlantic and Arctic Oceans (electronic supplementary material, S6). Based on the nature of the molecular characters (nuclear, co-dominant InDels) used for species identification and careful ground-truthing of our molecular results, we conclude that hybridization between the species, if it occurs at all, is rare or episodic.

## 4. Conclusion

Marine zooplankton have been regarded as sentinels of climate change [25] due to their short life histories and rapid responses to environmental variation. Development and use of molecular characters that can ensure accurate and reliable identification and discrimination of key indicator species, such as those within the *Calanus* genus, are critically needed. Only then can these species be used to document past, present and future patterns of biogeographical distributions, and detect and track responses of pelagic communities to climate change.

## Supplementary Material

Electronic Supplementary Material

## Supplementary Material

Supplementary 3

## Supplementary Material

Supplementary 4

## Data accessibility

Protocols are attached as the electronic supplementary material; genotypes and sequences have been deposited to public database, respectively Dryad (http://dx.doi.org/10.5061/dryad.tq71j) [26] and GenBank^®^ (MF959702-MF959730 and MF972920-MF972922).
