# Supplementary material for: Genetics redraws pelagic biogeography of Calanus
Source: Biol Lett. 2017 Dec 20;13(12):20170588. doi: 10.1098/rsbl.2017.0588 (PMC5746537; doi:10.1098/rsbl.2017.0588)
Supplement: Electronic Supplementary Material [file rsbl20170588supp1.pdf]

Electronic Supplementary Material, Choquet et al.
From: Genetics redraws pelagic biogeography of *Calanus*

Marvin Choquet[1*], Maja Hatlebakk[1,2], Anusha KS Dhanasiri[1], Ksenia Kosobokova[3], Irina Smolina[1], Janne E Søreide[2], Camilla Svensen[4], Webjørn Melle[5], Sławomir Kwaśniewski[6], Ketil Eiane[1], Malin Daase[4], Vigdis Tverberg[1], Stig Skreslet[1], Ann Bucklin[7], Galice Hoarau[1]

[1]*Faculty of Biosciences and Aquaculture, Nord University, Bodø, Norway*
[2]*Department of Arctic Biology, The University Centre in Svalbard, Longyearbyen, Norway*
[3]*Institute of Oceanology, Russian Academy of Sciences, Moscow, Russia*
[4]*Department of Arctic and Marine Biology, UiT-the Arctic University of Norway, Tromsø, Norway*
[5]*Institute of Marine Research, Bergen, Norway*
[6]*Institute of Oceanology, Polish Academy of Sciences, Sopot, Poland*
[7]*University of Connecticut, Groton, USA*

Supplementary1: Sampling information and molecular-based *Calanus* species composition within samples

Supplementary2: Phylogenetic relationships among 16S rDNA individual sequences of *Calanus finmarchicus*, *C. glacialis*, *C. hyperboreus* and *C. helgolandicus*

Supplementary3 (separate excel file): Test of congruence of *Calanus* species identification between 6 nuclear InDel markers and mitochondrial 16S rDNA, for individuals from the North Atlantic and Arctic Oceans

Supplementary4 (separate excel file): Test of congruence of *Calanus* species identification between 6 nuclear InDel markers and mitochondrial 16S rDNA for *Calanus finmarchicus* (*Cfin*) and *C. glacialis* (*Cgla*) in Saltenfjord/Skjerstadfjord accompanied with prosome length measurements and developmental stage information

Supplementary5: Frequency distributions of prosome length for *Calanus glacialis* and *C. finmarchicus* at developmental stage (**a**) CV and (**b**) adult female in the region of Saltenfjord / Skjerstadfjord

Supplementary6: Genotype admixture analysis based on nuclear InDels shows no hybrids between *Calanus finmarchicus* and *C. glacialis*

Supplementary7: Supplementary Protocols

Supplementary8: Sources of literature used for tracing the morphologically based distribution ranges of *Calanus* species

References of the Supplementary Material

SUPPLEMENTARY 1

Sampling information and molecular-based *Calanus* species composition within samples. N individuals per sample were genotyped with nuclear InDel markers. Samples relative proportions of *C. glacialis* (*Cgla*), *C. finmarchicus* (*Cfin*), *C. hyperboreus* (*Chyp*) and *C. helgolandicus* (*Chel*) are reported in percentage. In the Laptev Sea and the Nansen Basin, only species presence (x) or absence (-) is reported.

Locations	GPS	Date	Depth	N	%*Cgla*	%*Cfin*	%*Chyp*	%*Chel*	Collector
North Pole	89°43 N 14°11 E	4/9/2012	100-0 m	48	98%	0%	2%	0%	J. Søreide
Nansen Basin	87° N 55°47 E	10/4/2016	4400-0 m	94	x	x	-	-	K. Kosobokova
Ice West Svalbard	81°22 N 4°27 E	10/1/2012	200-0 m	48	25%	71%	4%	0%	I. Smolina
Ice North Svalbard	80°58 N 14°41 E	1/15/2016	200-0 m	44	9%	91%	0%	0%	M. Choquet
West Greenland 1	78°42 N 70°43 W	9/6/2013	100-0 m	48	40%	0%	60%	0%	J. Søreide
Laptev Sea	78°30 N 113°01 E	9/6/2013	100-0 m	91	x	x	x	-	E. Abramova
Isfjord	78°19 N 15°09 E	6/5/2016	20-0 m	138	64%	35%	1%	0%	M. Hatlebakk
West Greenland 2	77°51 N 71°49 W	9/9/2013	95-0 m	48	6%	0%	94%	0%	J. Søreide
Van Mijenfjord	77°46 N 15°02 E	6/3/2016	20-0 m	91	86%	13%	1%	0%	M. Hatlebakk
Chukchi Sea	76°24 N 162°14 W	7/27/2016	300-0 m	60	45%	0%	55%	0%	A. Bucklin
West Greenland 3	76°23 N 84°30 W	9/15/2013	100-0 m	48	38%	0%	63%	0%	J. Søreide
Bjørnøya 1	74°50 N 18°51 E	08/08/2012	50-0 m	48	67%	33%	0%	0%	I. Smolina
Bjørnøya 2	74°30 N 9°01 E	8/9/2013	200-0 m	47	0%	100%	0%	0%	I. Smolina
Fugløya-Bjørnøya	72°59 N 19°27 E	8/7/2013	100-0 m	47	0%	100%	0%	0%	I. Smolina
Porsangerfjord 1	70°43 N 25°44 E	8/21/2013	150-0 m	46	0%	100%	0%	0%	F. Norrbin
Porsangerfjord 2	70°40 N 25°39 E	6/3/2016	120-0 m	48	0%	100%	0%	0%	C. Svensen
Off-Tromsø	70°34 N 19°14 E	1/8/2016	300-0 m	79	6%	79%	0%	15%	M. Choquet
Lopphavet	70°25 N 21°50 E	6/2/2016	200-0 m	48	8%	92%	0%	0%	F. Norrbin
Østerbotn	70°06 N 25°09 E	8/25/2013	100-0 m	39	8%	90%	2%	0%	F. Norrbin
Gimsøy 1	69°57 N 9°35 E	8/5/2013	200-0 m	47	0%	100%	0%	0%	I. Smolina
Balsfjord 1	69°21 N 19°13 E	5/31/2016	170-0 m	39	90%	8%	2%	0%	C. Svensen
Balsfjord 2	69°21 N 19°13 E	5/3/2016	170-0 m	46	28%	52%	20%	0%	C. Svensen
Gimsøy 2	69°2 N 12°17 E	8/5/2012	200-0 m	46	0%	100%	0%	0%	I. Smolina
North Iceland	68°48 N 18°23 W	5/12/2013	200-0 m	48	0%	6%	94%	0%	I. Smolina
Tysfjord	68°07 N 16°11 E	4/9/2016	200-0 m	37	5%	60%	35%	0%	F. Norrbin
Vestfjord 1	68°06 N 14°28 E	5/30/2016	270-0 m	49	12%	76%	10%	2%	M. Krogstad
Vestfjord 2	68°05 N 14°58 E	4/10/2016	200-0 m	43	12%	21%	67%	0%	F. Norrbin
Vestfjord 3	68°02 N 14°27 E	6/20/2016	290-0 m	48	8%	90%	2%	0%	M. Krogstad
Sørfoldfjord 1	67°35 N 14°50 E	4/20/2016	510-0 m	43	51%	42%	7%	0%	M. Krogstad
Sørfoldfjord 2	67°34 N 15°11 E	4/20/2016	460-0 m	48	67%	29%	4%	0%	M. Krogstad
Sørfoldfjord 3	67°30 N 15°28 E	4/20/2016	480-0 m	55	76%	24%	0%	0%	M. Krogstad
Mistfjord 1	67°27 N 14°51 E	10/29/2013	290-0 m	40	25%	70%	5%	0%	M. Krogstad
Mistfjord 2	67°27 N 14°50 E	2/17/2016	285-0 m	82	45%	42%	13%	0%	M. Krogstad
Saltfjord 1	67°16 N 14°38 E	2/15/2016	375-0 m	98	42%	40%	18%	0%	M. Krogstad
Saltfjord 2	67°16 N 14°38 E	7/6/2016	360-0 m	93	30%	69%	1%	0%	M. Krogstad
Skjerstadfjord 1	67°15 N 14°50 E	7/12/2016	500-0 m	89	33%	50%	17%	0%	M. Krogstad
Skjerstadfjord 2	67°11 N 15°26 E	2/26/2016	385-0 m	151	61%	13%	26%	0%	M. Krogstad
Myken 1	67°03 N 13°31 E	8/3/2016	200-0 m	48	2%	96%	2%	0%	M. Krogstad
Myken 2	66°48 N 12°32 E	8/3/2016	210-0 m	45	0%	100%	0%	0%	M. Krogstad
Myken 3	66°46 N 12°30 E	8/3/2016	95-0 m	12	0%	100%	0%	0%	M. Krogstad
Myken 4	66°46 N 12°28 E	8/3/2016	28-0 m	11	0%	73%	0%	27%	M. Krogstad
Myken 5	66°46 N 12°23 E	8/3/2016	140-0 m	12	0%	100%	0%	0%	M. Krogstad
Myken 6	66°45 N 12°28 E	8/3/2016	95-0 m	12	0%	100%	0%	0%	M. Krogstad
North Atlantic 1	66°43 N 7°46 W	5/8/2013	1000-0 m	46	7%	80%	13%	0%	I. Smolina
White Sea	66°33 N 33°43 E	8/22/2016	100-0 m	115	100%	0%	0%	0%	K. Kosobokova
Ranfjord 1	66°17 N 14°00 E	6/14/2016	410-0 m	47	98%	2%	0%	0%	M. Krogstad
Ranfjord 2	66°14 N 13°24 E	6/14/2016	275-0 m	46	63%	37%	0%	0%	M. Krogstad
Ranfjord 3	66°12 N 12°49 E	6/14/2016	215-0 m	45	13%	87%	0%	0%	M. Krogstad
North Atlantic 2	65°03 N 00°51 W	5/5/2013	1000-0 m	48	4%	88%	8%	0%	I. Smolina
South Iceland	63°49 N 24°18 W	5/14/2013	200-0 m	48	0%	100%	0%	0%	I. Smolina
Steinkjer	63°49 N 11°18 E	6/14/2016	50-0 m	45	0%	98%	0%	2%	G. Hoarau & K. Eiane
North Atlantic 3	63°45 N 02°16 E	5/4/2013	200-0 m	47	0%	100%	0%	0%	I. Smolina
Trondheimsfjord	63°29 N 10°14 E	8/22/2016	450-0 m	48	4%	96%	0%	0%	Ø. Leiknes
North Atlantic 4	62°50 N 28°17 W	5/15/2013	1000-0 m	40	0%	95%	5%	0%	I. Smolina
North Atlantic 5	62°50 N 02°31 W	4/13/2012	150-0 m	48	2%	98%	0%	0%	S. Basedow
Svinøy	62°37 N 05°20 E	01/08/12	127-0 m	51	0%	86%	0%	14%	I. Smolina
Labrador 1	62°13 N 57°21 W	5/26/2013	1000-0 m	95	3%	86%	11%	0%	I. Smolina
North Atlantic 6	61°30 N 10°59 W	4/10/2012	50-0 m	48	0%	100%	0%	0%	S. Basedow
Sognefjord	61°11 N 06°35 E	6/22/2016	200-0 m	41	0%	15%	0%	85%	T. Dale
Lurefjord 1	60°43 N 05°04 E	6/22/2016	230-0 m	48	81%	19%	0%	0%	M. Choquet
Lurefjord 2	60°41 N 05°09 E	6/22/2016	340-0 m	92	88%	12%	0%	0%	M. Choquet
Lurefjord 3	60°37 N 05°11 E	6/22/2016	160-0 m	49	90%	8%	0%	2%	M. Choquet
Christianssund	60°36 N 38°20 W	6/1/2013	1000-0 m	45	0%	93%	7%	0%	I. Smolina
Osterfjord	60°34 N 05°24 E	6/23/2016	550-0 m	47	4%	83%	4%	9%	M. Choquet
Sørfjord 1	60°31 N 05°21 E	6/23/2016	487-0 m	48	0%	94%	6%	0%	M. Choquet
Sørfjord 2	60°28 N 05°40 E	6/23/2016	360-0 m	43	0%	81%	0%	19%	M. Choquet
Sørfjord 3	60°26 N 05°30 E	6/23/2016	225-0 m	48	0%	79%	0%	21%	M. Choquet
North Sea	60°20 N 01°00 E	4/2/2012	50-0 m	45	0%	76%	0%	24%	S. Basedow
Raunefjord	60°17 N 05°08 E	6/4/2016	180-0 m	45	0%	98%	2%	0%	W. Melle
Korsfjord	60°11 N 05°12 E	6/6/2016	200-0 m	48	0%	94%	0%	6%	W. Melle
Hardangerfjord	59°58 N 05°41 E	8/30/2016	106-0 m	46	0%	48%	0%	52%	A. Mailli
Labrador 2	59°47 N 52°16 W	5/28/2013	1000-0 m	47	0%	91%	9%	0%	I. Smolina
Oslofjord	59°12 N 10°38 E	4/16/2016	350-0 m	51	0%	63%	31%	6%	T. Falkenhaug
Lenefjord	58°5 N 07°9 E	7/18/2016	220-0 m	45	0%	56%	11%	33%	K. Eiane
Risørfjord	58°44 N 09°15 E	4/15/2016	160-0 m	47	0%	87%	0%	13%	T. Falkenhaug
Grønsfjord	58°2 N 07°2 E	7/18/2016	190-0 m	48	0%	75%	8%	17%	K. Eiane
Loch Etive	56°27 N 05°11 W	9/19/2016	130-5 m	47	0%	100%	0%	0%	K. Last
Off-Ainort	56°51 N 05°53 W	7/31/2008	20-0 m	48	0%	4%	0%	96%	S. Wells
Quebec 1	50°26 N 50°42 W	5/11/2014	200-0 m	47	0%	77%	23%	0%	A. Bucklin
Quebec 2	45°05 N 53°44 W	5/15/2014	200-0 m	47	4%	70%	26%	0%	A. Bucklin
US Portland	42°59 N 68°52 W	8/22/2012	200-0 m	47	0%	100%	0%	0%	A. Bucklin
US New-York	40°19 N 69°50 W	8/14/2012	200-0 m	45	0%	100%	0%	0%	A. Bucklin
US Philadelphia	39°58 N 72°46 W	8/8/2012	200-0 m	51	0%	100%	0%	0%	A. Bucklin

SUPPLEMENTARY 2

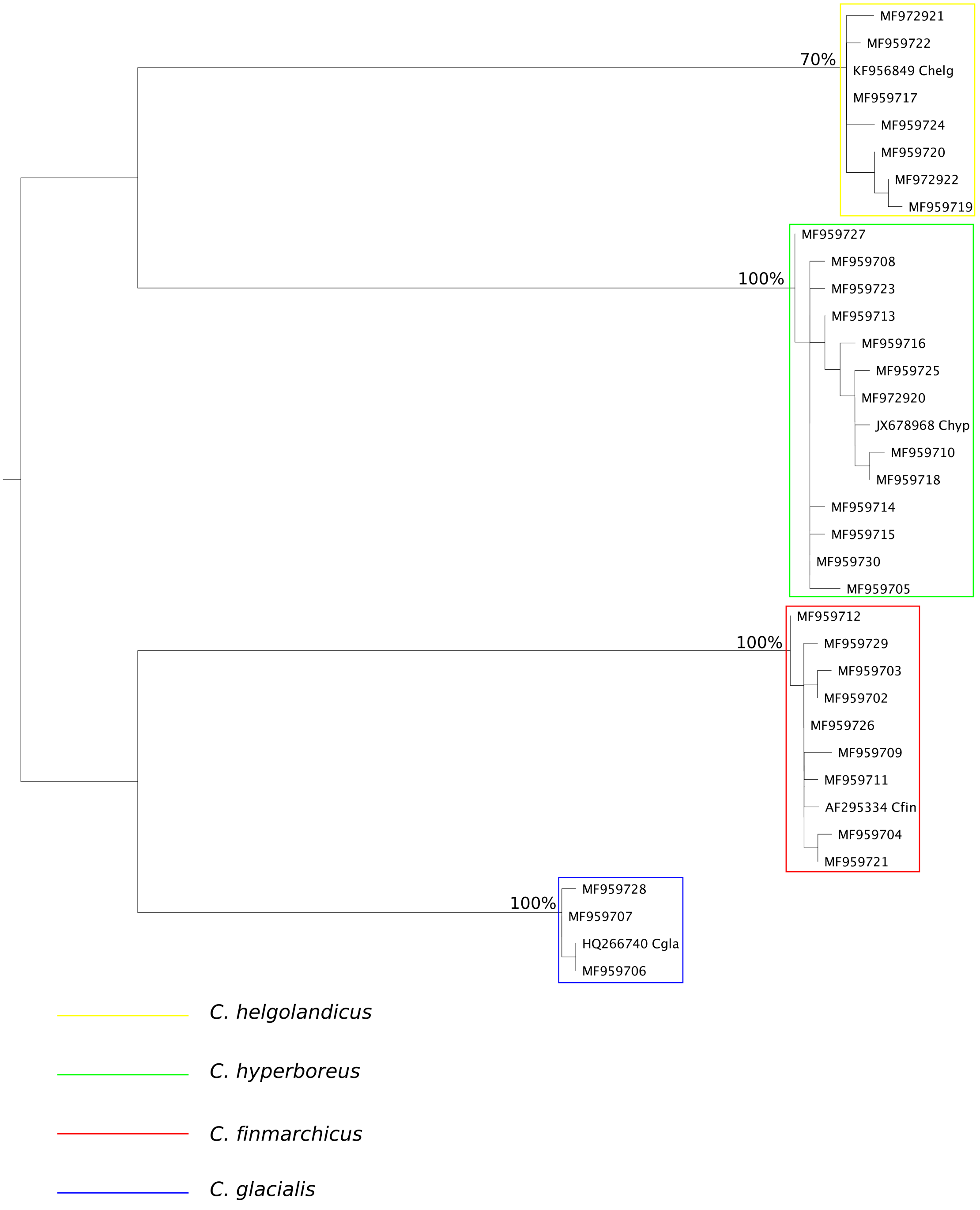

Phylogenetic relationships among 16S rDNA individual sequences of *Calanus finmarchicus*, *C. glacialis*, *C. hyperboreus* and *C. helgolandicus*

SUPPLEMENTARY 5

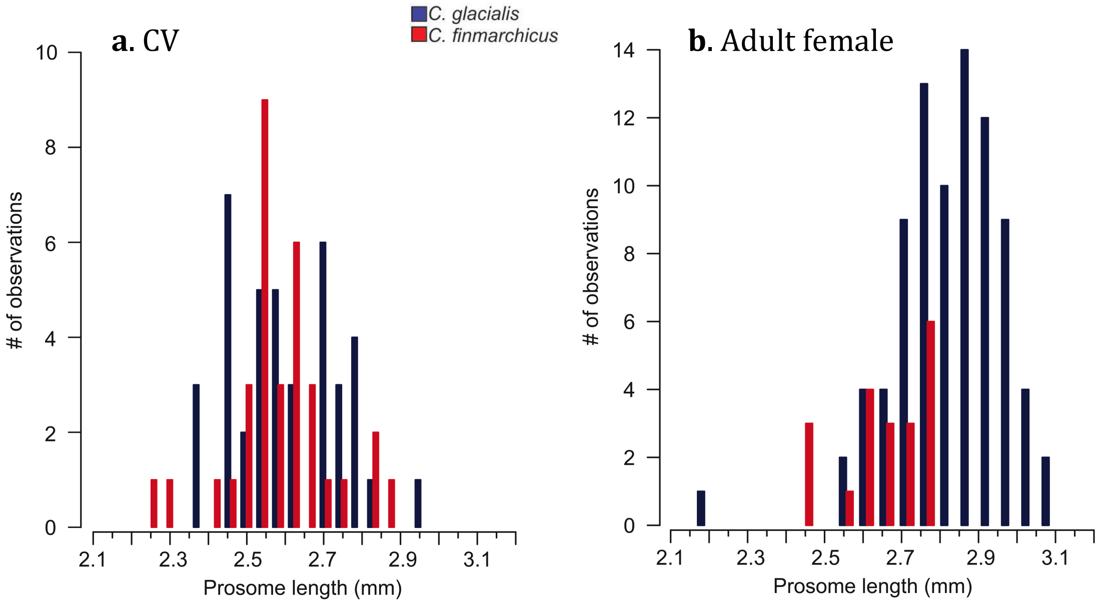

Frequency distributions of prosome length for *Calanus glacialis* and *C. finmarchicus* at developmental stage (a) CV and (b) adult female in the region of Saltenfjord / Skjerstadfjord

In total, prosome length of 171 *Calanus* individuals was measured.

SUPPLEMENTARY 6

Genotype admixture analysis based on nuclear InDels shows no hybrids between *Calanus finmarchicus* and *C. glacialis*

Bar chart representing genotype admixture analysis results based on nuclear InDel genotypes data, performed using STRUCTURE (v. 2.3.4) after Nielsen *et al.,* 2014[1].

- **a.** Results from the first set of InDels used (c.f. Supplementary7),
- **b.** Results from the second set of InDels.

Each individual is represented by a bar filled with one or two distinct colours that identify an individual probability to belong to two clusters (here, green for *C. finmarchicus* and red for *C. glacialis*). In case of F1 hybrids between *C. finmarchicus* and *C. glacialis*, a bar will be nearly equally filled with both colours (which never happened).

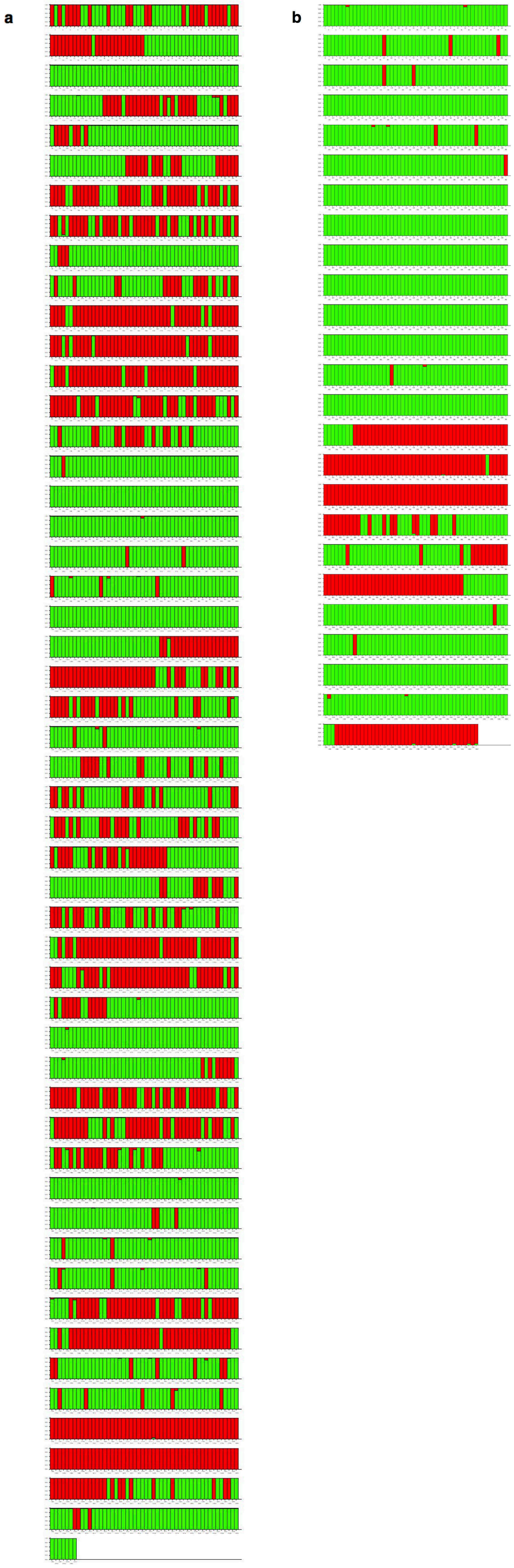

SUPPLEMENTARY 7

Supplementary Protocols

- DNA extraction:

We extracted DNA from the antennules of each specimen, using the quick and cheap method of HotSHOT DNA extraction[2]:

1- Individuals were soaked separately in sterile water to rinse the ethanol;
2- One by one, under a stereomicroscope, the 2 antennules were removed from the rest of the body and placed in 50 µL of a Lysis Buffer (See HotSHOT protocol for details about composition of buffers[2]) in a 96-well plate;
3- The plate was incubed in a thermocycler, 30 minutes at 95°C;
4- The plate was subsequently cooled in the fridge (4°C) for 5-10 minutes;
5- Finally, 50 µL of Neutralizing Solution was added (See HotSHOT protocol for details about composition of buffers[2]).

- Molecular species identification:

We amplified a set of 6 nuclear molecular markers, type InDel (polymorphism consists of Insertion or Deletion of nucleotides): G_150, T_461, T_1338, T_1966, T_3133 and T_4700[3] in a single multiplexed Polymerase Chain Reaction (PCR), and genotyped them following the protocol described by Smolina *et al.* (2014)[3]. Four distinct patterns of genotypes were distinguished and assigned to the four different species of *Calanus* based on species-specific alleles defined in Smolina *et al.* (2014)[3]. This method is fast and inexpensive. A total of 96 individuals can be reliably identified within 5 hours, with 100% reliable results for ca. 2 euros/sample. At one point in our study, we had to re-order a new stock of InDel primers from a new provider, and thus had to change the type of fluorescent dye labelling of the forward primers (from 6-FAM, VIC and NED (Life Technologies) to FAM, YAKYE and ATTO550 (Eurofins Genomics)). This resulted in a slight shift of the length of the alleles in the genotyping, thus this second set of data was treated separately. To confirm the species identification, and in order to validate our nuclear markers, we sequenced a portion of the mitochondrial 16S rDNA (ca. 360bp)[4] for 159 individuals from 53 locations selected to represent the full range of sampling, and for 129 individuals from the region of Saltenfjord / Skjerstadfjord, following the same protocol described in Smolina *et al.* (2014)[3]. The obtained sequences were then aligned together with one reference 16S sequence for each species from GenBank®: HQ266740 for *C. glacialis*, AF295334 for *C. finmarchicus*, KF956849 for *C. helgolandicus*, and JX678968 for *C. hyperboreus*. This alignment was used to reconstruct a PhyML tree (GTR model) using Geneious version 9.1 (http://www.geneious.com)[5]. The resulting tree displayed four clearly distinct groups of sequences corresponding to the four species (see Supplementary2). In all individuals, this approach resulted in the same species identification as the InDel genotyping (Supplementary3-4).

- Microsatellite analysis:

To characterize connectivity among newly described population of *C. glacialis* in Norwegian fjords and other regions and compare it to *C. finmarchicus* we performed analysis of population genetic differentiation using sample from 3 locations: Isfjord, Saltfjord and Lurefjord (c.f. Supplementary1). DNA from the antennas of 24 identified (InDels method – see above) individuals per species and per location was used to amplify 10 microsatellites markers[6, 7] by PCR. Nine microsatellite markers were multiplexed into 3 PCR reactions (EL696609, EL585922, and EL773519; FK868270, FG632811, and FK670364; EH666870, EH666474, and EL773359) and one marker, FK867682, was amplified separately. PCR reactions were carried out in a final volume of 5 µL, using 2.5 µL of Accu-Start Tough Mix® (Quanta Biosciences), 0.1 µL of each primer at 10 µM, and completing with DNA from HotSHOT extraction. Reactions were run on a Veriti® 96-well fast thermal cycler (Applied Biosystems). Amplification reactions consisted of Multiplex 1 and FK867682 for 3 min at 95°C; with 35 cycles of: 95°C (30 sec), 55°C (60 sec), and 72°C (60 sec); with one final extension cycle at 72°C for 5 min. Multiplex 2 was amplified by a first cycle at 95°C (3 min), followed by 35 cycles of: 95°C (30 sec), 53°C (60 sec), and 72°C (60 sec); and one final cycle at 72°C (5 min) for extension. Multiplex 3 was run as 95°C (3 min), and then 10 cycles of: 95°C (1 min), touch-down from 68°C (1 min) to 58°C (1 min), and 72°C (1 min); followed by 25 cycles of: 95°C (1 min), 58°C (1 min), and 72°C (1 min); and a final extension cycle at 72°C (5 min). Microsatellite loci were analysed on a 3500XL Genetic Analyzer (Life Technologies) in three multiplex and one singleplex following Nielsen *et al.* (2014)[8].
We used *Genetix* (v. 4.05.2)[9] to estimate the global F_{ST}, index of population differentiation after Weir & Cockerham[10].

SUPPLEMENTARY 8

Sources of literature used for tracing the morphologically based distribution ranges of *Calanus* species

The map showing the distribution ranges of *Calanus* species in the North Atlantic and Arctic Ocean, as defined from morphological identification of species, presented as Figure 1 of the paper, was mainly based on three different sources: Conover, 1988[11], Barnard et al., 2004[12] for the southern borders of species distributions, and Jaschnov, 1970[13] for the northern borders. Additional complementary sources were: Wassmann et al., 2015[14]; Melle et al., 2014[15]; Estrada et al., 2012[16]; Bonnet et al., 2005[17].

References:

[1] Pritchard, J.K., Stephens, M. & Donnelly, P. 2000 Inference of population structure using multilocus genotype data. *Genetics* **155**, 945-959.
[2] Montero‐Pau, J., Gómez, A. & Muñoz, J. 2008 Application of an inexpensive and high‐throughput genomic DNA extraction method for the molecular ecology of zooplanktonic diapausing eggs. *Limnology and Oceanography: Methods* **6**, 218-222.
[3] Smolina, I., Kollias, S., Poortvliet, M., Nielsen, T.G., Lindeque, P., Castellani, C., Moller, E.F., Blanco-Bercial, L. & Hoarau, G. 2014 Genome- and transcriptome-assisted development of nuclear insertion/deletion markers for *Calanus* species (Copepoda: Calanoida) identification. *Molecular ecology resources* **14**, 1072-1079. (doi:10.1111/1755-0998.12241).
[4] Sanger, F. & Coulson, A.R. 1975 A rapid method for determining sequences in DNA by primed synthesis with DNA polymerase. *Journal of molecular biology* **94**, 441IN19447-19446IN20448.
[5] Kearse, M., Moir, R., Wilson, A., Stones-Havas, S., Cheung, M., Sturrock, S., Buxton, S., Cooper, A., Markowitz, S. & Duran, C. 2012 Geneious Basic: an integrated and extendable desktop software platform for the organization and analysis of sequence data. *Bioinformatics* **28**, 1647-1649.
[6] Parent, G.J., Plourde, S. & Turgeon, J. 2012 Natural hybridization between *Calanus finmarchicus* and *C. glacialis* (Copepoda) in the Arctic and Northwest Atlantic. *Limnology and Oceanography* **57**, 1057-1066. (doi:10.4319/lo.2012.57.4.1057).
[7] Provan, J., Beatty, G.E., Keating, S.L., Maggs, C.A. & Savidge, G. 2009 High dispersal potential has maintained long-term population stability in the North Atlantic copepod *Calanus finmarchicus*. *Proceedings. Biological sciences / The Royal Society* **276**, 301-307. (doi:10.1098/rspb.2008.1062).
[8] Nielsen, T.G., Kjellerup, S., Smolina, I., Hoarau, G. & Lindeque, P. 2014 Live discrimination of *Calanus glacialis* and *C. finmarchicus* females: can we trust phenological differences? *Marine Biology* **161**, 1299-1306. (doi:10.1007/s00227-014-2419-5).
[9] Belkhir, K., Borsa, P., Chikhi, L., Raufaste, N. & Bonhomme, F. 1996 GENETIX 4.05, logiciel sous Windows TM pour la génétique des populations. *Laboratoire génome, populations, interactions, CNRS UMR* **5000**, 1996-2004.
[10] Weir, B.S. & Cockerham, C.C. 1984 Estimating F-statistics for the analysis of population structure. *Evolution*, 1358-1370.
[11] Conover, R. 1988 Comparative life histories in the genera *Calanus* and *Neocalanus* in high latitudes of the northern hemisphere. In *Biology of Copepods* (pp. 127-142, Springer.
[12] Barnard, R., Batten, S., Beaugrand, G., Buckland, C., Conway, D., Edwards, M., Finlayson, J., Gregory, L., Halliday, N. & John, A. 2004 Continuous plankton records: Plankton atlas of the North Atlantic Ocean (1958-1999). II. Biogeographical charts. *Marine Ecology-Progress Series*, 11-75.
[13] Jaschnov, W. 1970 Distribution of *Calanus* species in the seas of the northern hemisphere. *Internationale Revue der gesamten Hydrobiologie und Hydrographie* **55**, 197-212.
[14] Wassmann, P., Kosobokova, K., Slagstad, D., Drinkwater, K., Hopcroft, R., Moore, S., Ellingsen, I., Nelson, R., Carmack, E. & Popova, E. 2015 The contiguous

domains of Arctic Ocean advection: trails of life and death. *Progress in Oceanography* **139**, 42-65.
[15] Melle, W., Runge, J., Head, E., Plourde, S., Castellani, C., Licandro, P., Pierson, J., Jonasdottir, S., Johnson, C. & Broms, C. 2014 The North Atlantic Ocean as habitat for *Calanus finmarchicus*: Environmental factors and life history traits. *Progress in Oceanography* **129**, 244-284.
[16] Estrada, R., Harvey, M., Gosselin, M., Starr, M., Galbraith, P.S. & Straneo, F. 2012 Late-summer zooplankton community structure, abundance, and distribution in the Hudson Bay system (Canada) and their relationships with environmental conditions, 2003–2006. *Progress in oceanography* **101**, 121-145.
[17] Bonnet, D., Richardson, A., Harris, R., Hirst, A., Beaugrand, G., Edwards, M., Ceballos, S., Diekman, R., López-Urrutia, A. & Valdes, L. 2005 An overview of *Calanus helgolandicus* ecology in European waters. *Progress in Oceanography* **65**, 1-53.
